# Safety of Innovative Nanotechnology Oral Formulations Loaded with Bioactive Menopause Molecules: Influence of Genotoxicity and Biochemical Parameters on a Menopausal Rat Model

**DOI:** 10.3390/nu15234951

**Published:** 2023-11-29

**Authors:** Simona Crișan, Anca Lucia Pop, Ioana Lacatusu, Nicoleta Badea, Cosmin Mustaciosu, Mihai Radu, Valentin Nicolae Varlas, Ovidiu Nicolae Peneş, Anne Marie Ciobanu, Manuela Ghica, Sorina Nicoleta Voicu, Denisa Ioana Udeanu

**Affiliations:** 1 Faculty of Pharmacy, “Carol Davila” University of Medicine and Pharmacy, 6 Traian Vuia Street, 020945 Bucharest, Romania; simona.oprescu@drd.umfcd.ro (S.C.); anca.pop@umfcd.ro (A.L.P.); anne.ciobanu@umfcd.ro (A.M.C.); manuela.ghica@umfcd.ro (M.G.); denisa.udeanu@umfcd.ro (D.I.U.); 2 R&D Center, AC HELCOR, Victor Babes St., 430082 Baia Mare, Romania; 3 Faculty of Applied Chemistry and Materials Science, The Polytechnic University of Bucharest, Polizu No 1, 011061 Bucharest, Romania; ioana.lacatusu@upb.ro (I.L.); nicoleta.badea@upb.ro (N.B.); 4 Horia Hulubei National Institute for Physics and Nuclear Engineering IFIN-HH, 077125 Bucharest, Romania; cosmin@nipne.ro (C.M.); mradu@nipne.ro (M.R.); 5 Faculty of Medicine, “Carol Davila” University of Medicine and Pharmacy, 37 Dionisie Lupu Street, 020021 Bucharest, Romania; 6 Department of Biochemistry and Molecular Biology, Faculty of Biology, University of Bucharest, 030018 Bucharest, Romania; sorina.voicu@bio.unibuc.ro

**Keywords:** nano lipid matrix, nanotoxicology, preclinical studies, biochemical parameters, menopause model, nanostructured lipid carriers (NLCs), menopause disorder treatment, nano diosgenin, glycyrrhizic acid

## Abstract

In recent years, nanoparticles have gained significant importance due to their unique properties, such as pharmacological, electrical, optical, and magnetic abilities, contributing to the growth of the science and technology sector. Particular naturally derived biomolecules with beneficial effects on menopause disorder have been the subject of studies of pharmaceutical formulation to obtain alternative pharmaceutical forms with increased bioavailability and without side effects, as in nanostructured lipid carriers (NLCs) loaded with such active ingredients. In the present study, one stage of a broader project, we have performed pharmacotoxicology studies for six combinatory innovative nanocapsule pharmaceutical forms containing active natural biomolecules before considering them as oral formulas for (1) in vitro toxicity studies on culture cells and (2) in vivo preclinical studies on a surgically induced menopause model of Wistar female rats, and the influence of the NLCs on key biochemical parameters: lipid profile (TG, Chol, HDL), glycemic markers (Gli), bone markers (Pac, Palc, Ca, phosphorus), renal markers (Crea, urea, URAC), inflammation (TNF), oxidative stress (GSH, MDA), and estrogen–progesterone hormonal profile. The micronucleus test did not reveal the genotoxicity of the tested compounds; the menopause model showed no significant safety concerns for the six tested formulas evaluated using the blood biochemical parameters; and the results showed the potential hypoglycemic, hypolipidemic, hypouricemic, and antioxidant potential of one of the tested formulas containing nano diosgenin and glycyrrhizic acid.

## 1. Introduction

Menopause has long-term consequences on the health of the body, the early onset being accompanied by the exacerbation of clinical manifestations [1,2]. The most frequent adverse reactions occur due to imbalances in bone, lipid, or carbohydrate metabolism, favoring the appearance of osteoporosis and cardiovascular [3], liver, kidney, and neurological disturbances [4,5]. In the absence of adequate treatment and secondary to endocrine disorders, the quality of life of women is altered at a fully active age [6,7]. Progesterone and estrogen hormonal replacement therapy (HRT) in menopause is recommended to relieve symptoms. However, this approach has several contraindications, accompanied in the long term by an increased risk of coagulation disorders (200% increased risk of blood clots), myocardial infarction (29% increased risk), or breast cancer (8 additional cases per 10,000 women per year) [8,9]. Currently, certain biomolecules with beneficial effects in the adjuvant treatment of menopause [10,11] are the subject of studies of pharmaceutical formulation to obtain therapeutic alternatives with increased stability and bioavailability, a reduced dose of the active compound [12], and without or reduced secondary side effects [13,14].

Diosgenin (DSG, 3β-Hydroxy-5-spirostene), a steroidal sapogenin, exhibits anti-inflammatory, anti-cancer, and anti-diabetic activities by modulating multiple signaling pathways. Found in various plants, including fenugreek and wild yam, Dioscorea rhizome, Dioscorea septemloba, and Rhizoma polygonate, DSG is renowned for its biochemical activity that mimics or modulates estrogen, the primary female sex hormone due to its structural similarity to endogenous estrogens, enabling it to interact with estrogen receptors (ERs) [15,16]. When it binds to ERs, DSG can modulate the transcription of estrogen-responsive genes, altering the expression levels of proteins related to estrogenic signaling pathways, and so it may help regulate hormonal balance, proposing it as a candidate for an alternative to hormone replacement therapy (HRT), thereby ameliorating menopausal symptoms like hot flashes, mood swings, and osteoporosis in women [17,18]. The serotonin system is involved in regulating mood, temperature, and the hypothalamic–pituitary–gonadal axis, areas typically affected during menopause [19].

Glycyrrhizic acid from licorice root has diverse pharmacological effects: anti-inflammatory by inhibiting prostaglandin synthesis, antiviral against SARS-associated coronaviruses, hepatoprotective via cytochrome P450 2E1 suppression, and causing the modulation of cortisol activity by inhibiting 11β-hydroxysteroid dehydrogenase [20]. Black cohosh’s active ingredient, triterpene glycoside, alleviates menopausal symptoms potentially via serotonin receptors, offering an alternative to hormone therapy [21]. The triterpene glycosides in black cohosh may modulate the serotonin receptors in regulating mood, temperature, and the hypothalamic–pituitary–gonadal axis, areas typically affected during menopause [22,23].

The effectiveness of the oral administration of the active ingredients and biomolecules depends on the degree of their degradation in the severely acid environment of the stomach. The use of nanoparticles in recent years as a new scientific model has gained significant importance in various fields due to their unique properties, which contribute to the increased involvement of science and technology [24]. Nanomedicine has provided new approaches to improve oral delivery of drugs with poor solubility, low stability and bioavailability, and various administration restrictions [25]. Nanostructured lipid carriers (NLCs) are the latest-generation colloidal nanoparticles with improved stability and drug-loading efficiency. The lipids used in NLC preparation are usually physiological (biocompatible and biodegradable) [26]. Therefore, it has become vital to understand nanoparticles’ impact on human health and the environment [27].

The safety and biological interactions of nanomaterials are investigated using nanotoxicology. Thus, as nanotechnology advances, understanding the biological repercussions of nanoparticles becomes crucial. Preclinical nanotoxicology tests collect information on toxicity testing (single-dose and repeated-dose) of lipid nanoparticles [28] loaded with active pharmaceutical ingredients.

Laboratory rodents (rats, mice, rabbits) and carnivores are most commonly used in toxicity studies. The trend toward limiting animal experimentation has led to the development of “alternative methods” for toxicity testing on cell cultures, tissues, or embryos. However, these alternative methods cannot completely replace traditional live animal tests, with some disadvantages related to the culture survival time, the choice of revealing toxicity parameters, and the fact that cell culture is an isolated system. A preclinical, experimental model of menopause can be obtained in three ways: (1) using temporal (natural) aging (“model of aging menopausal rat females”) [29]; (2) pharmacological (chemical castration using ovotoxin) [30]; (3) surgical, via ovarectomy [30,31].

To date, there is a lack of safety studies on NLCs incorporating standardized plant extracts of wild yam (*Dioscorea villosa* L.), licorice (*Glycyrrhiza glabra*), and black cohosh (*Actaea racemosa*) regarding their safety and biochemical parameters since NLCs have reported effects on menopausal status [32]. The present study was designed to assess the safety of loaded nano lipid carrier formulations formulated and scaled up in previous research stages [33,34] as a part of a broader research study [35,36], determining the in vitro toxicity on cultured cells, i.e., the genotoxicity and safety of the formulations and the influence of treatment with different nanoparticle pharmaceutical formulations on laboratory parameters in a preclinical model of menopause [37,38].

## 2. Materials and Methods

**Figure 1:**
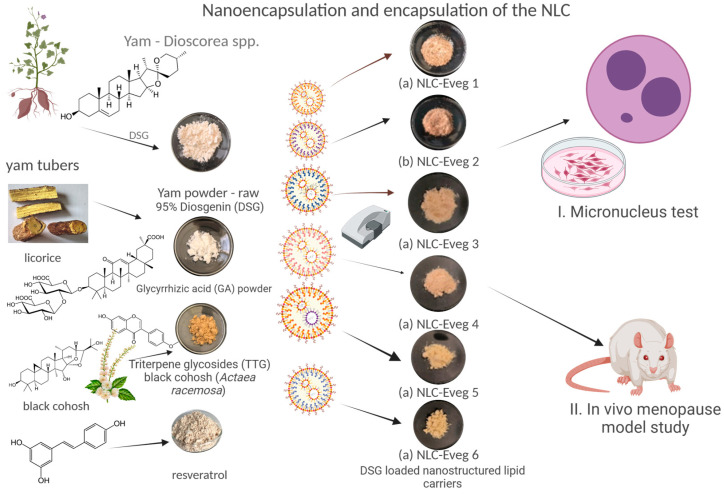
Study plan.

During the present study, as a part of broader research, we investigated the cytotoxicity of the tested NLCs using the micronucleus test on culture cells during the first stage. In the second part, we tested the influence of NLCs on the biochemical parameters of menopausal (surgically induced) Wistar female rats to (1) test the influence on the overall biochemical status lipid profile (TG, Chol, HDL), bone markers (Pac, Palc, Ca, phosphorus), glycemic markers (Gli), renal markers (Crea, urea, URAC), general status and body weight, inflammation parameters (TNF), and oxidative stress (GSH, MDA), and as the formulas target the hormonal load, (2) we have tested hormonal markers—progesterone and serum estradiol (Figure 1).

### 2.1. NLC Samples Taken for Study

The tested samples consist of plant extracts embedded into nanostructured lipid carriers (NLCs), prepared and evaluated in previous research stages [29,30].

The NLCs were loaded with single and combined herbal extracts using the melt emulsification technique (MET) and high-pressure homogenization (HPH), the procedure outlined in previous work conducted by our research team [39] based on (1) liquid oil phase evening primrose oil (EPO); soybean oil (SBO); flaxseed oil (FSO); and Silybum marianum oil (SMO); and (2) solid active ingredients: diosgenin (DSG); glycyrrhizic acid (GA); triterpene glycosides (TTG); resveratrol (PP) in different proportions, subject to a patent (Table 1 and Table 2). All the ingredients are approved for human use [33,34].

### 2.2. In Vitro Cell Culture Cytotoxicity Testing: Viability and the Micronucleus Test

The in vitro cytotoxicity test on culture cell tests was conducted at the Bioeval Laboratory (The National Institute of Physics and Nuclear Engineering—Horia Hulubei (IFIN-HH), Măgurele, Romania). The culture medium contained phenol red, and no changes in color were observed in the most concentrated samples; in the culture medium, they form precipitates visible under an inverted microscope with a magnification of 100×. A negative control, dimethyl sulfoxide (DMSO, Thermo Fischer Scientific, Waltham, MA USA), was chosen in a maximum concentration of 0.1% and used as a solvent for test samples, and methyl methanesulfonate (MMS, Sigma code 129925) as a positive control. All test samples were kept in the refrigerator (2–8 °C) until use. The samples were sterilized using gamma radiation [40] and kept sterile until use.

Cell line. The test was conducted on Chinese hamster pulmonary fibroblasts—V79 from ATCC, one of the cell lines recommended by OECD 487 paragraph 14. During the work, the cell culture was monitored by cell counting and visualized under an inverted microscope of the attached cells. The absence of mycoplasma in cell culture was confirmed using the MycoAlert^®^ Mycoplasma Detection Kit (Lonza code LT07-318). The cells have a doubling time of approximately 18 h, to which the procedures in this test are reported.

#### 2.2.1. Determination of Viability

The cytotoxicity was determined according to ISO 10993-5:2014 Annex C by measuring the metabolic activity of the cells after contact with the test product [41]. MTT (3-(4,5-dimethylthiazol-2-yl)-2,5-diphenyl tetrazolium bromide) (Serva code 20395) is yellow, water-soluble and metabolically reduced in viable cells into formazan, which is insoluble and blue–violet in color. The number of viable cells is correlated with the color intensity determined using photometric measurements after the formazan has dissolved in the DMSO. The cells were seeded at a density of 10,000/well, and after 24 h of culture, the test product was added in different concentrations. After 24 h of incubation with the test product, the medium was removed, the MTT solution was added to incubate for 3 to 4 h, and then the medium was removed and dissolved with DMSO. The absorbent was then read at 570 nm.

#### 2.2.2. Determination of Genotoxicity

The in vitro testing of the genotoxicity of plant extracts using the micronucleus formation method involves cell growth in the presence of plant extracts and the evaluation of genotoxic lesions by counting the micronuclei formed in the cell.

Cytochalasin B (cytoB Sigma code C6762) at a concentration of 5 μg/mL in a growth medium for 24 h was used to block cytokinesis. The cytoB stock was dissolved in DMSO. The sample test concentration was calculated according to paragraph 30 of OECD 487 because it is considered a less soluble substance that is not cytotoxic at a concentration lower than the lowest insoluble concentration. Thus, the highest concentration analyzed was the one at which precipitates were observed under the inverted microscope.

The cells were cultured in DMEM (Gibco code 11965084) supplemented with 10% bovine fetal serum (Sigma code F7524) and a combination of 100 IU/mL penicillin with 100 μg/mL streptomycin (Biological Industries code 03-031-1B) grown in the incubator at 37 °C, with 5% carbon dioxide in the air and a relative humidity of about 95%. After 28 h, the cells were fixed with 3.7% formaldehyde (Chemical Company code 605-001-00-5), stained with acridine orange (Sigma code A-6014), and examined under a fluorescence microscope (Olympus BX51, Tokyo, Japan). After long exposure to the environment, cytoB was added to the test substance and incubated for 24 h, after which the cells were prepared as before.

**Figure 2:**
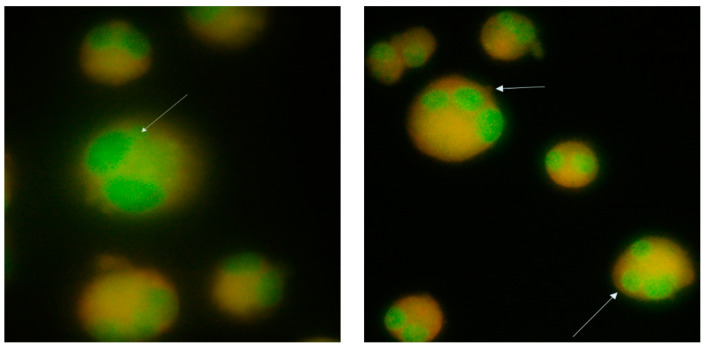
Cells stained with orange acryline (orange cytoplasm, green nucleus marked by arrows), (**left**) binucleated cell with micronucleus, (**right**) cells with three nuclei.

Methyl methanesulfonate (MMS, Sigma Aldrich code 129925) was used as a positive control at 5 and 10 μM concentrations. We counted the micronuclei meeting the following cumulative criteria: (a) the diameter was less than one-third of the central nucleus but large enough to discern its shape and color; (b) had a similar texture and coloration to the main nucleus and was in the same focal plane as the nucleus; (c) had a smooth, rounded perimeter suggesting a membrane; (d) was separated or overlapped marginally with the main nucleus as long as there was a clear identification of the nuclear boundary (Figure 2).

For the screening of the microscope slides, the zigzag method was used without passing through the same area twice; for each sample, we counted 2000 binucleate cells. MTT assay was used to measure the cytotoxicity.

### 2.3. In Vivo Tests on a Female Model of Menopausal Rats

**Figure 3:**
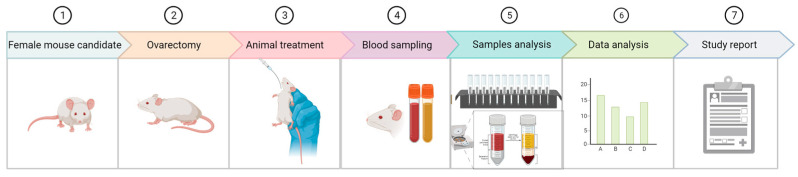
Representation of the in vivo preclinical study flow (designed with Biorender^®^, 2023).

This stage of the study was designed using literature articles [42,43] and animal welfare standards and laws [44,45,46] (Figure 3). The study aimed to determine the influence of treatment using different pharmaceutical formulations based on the nanoparticles of diosgenin and resveratrol in a suspension of 10% concentration in ovariectomized female Wistar rats in a preclinical, experimental model of the induction of menopause (Ethics Committee approval 100CECP/9.02.2022, IFIN-HH, and approval from the National Veterinary and Food Safety Authority Ilfov No. 29/22.02.2022, Romania).

We tested female Wistar rats from an authorized breeder (“Cantacuzino”National Institute for Medical Military Research and Development, Bucharest, Romania). The animals were kept for at least five days in quarantine and five days in accommodation cages to accommodate the conditions of the new environment. The feed consisted of granulated compound feed for mice, rats, and hamsters according to Declaration of Conformity No. 38/17.03.2022.

Food and water were available ad libitum throughout, with the animals deprived of food only 24 h before surgery to provide proper anesthesia and reduce pressure on the abdominal walls. The animals were divided into homogeneous groups of eight individuals, to which the same compound was administered. Throughout the experiment, the animals were accommodated in individual cages, the bedding was changed every two to three days, the temperature was between 19 and 23 °C, and the relative humidity in the air was between 40 and 60%. The tested subjects were observed for general appearance, behavioral changes, and mortality for the first 30 min, 4 h, 24 h, and then once daily for 14 days.

#### 2.3.1. Induced Menopause Model

We chose oophorectomy as the method of inducing menopause as the “golden standard” for modeling menopause, as menopause induced by oophorectomy is predominantly used in research compared to induction by aging, and the duration of the study on a menopause model induced by aging would last over 10 months [47]. The veterinarian performed the surgery under aseptic conditions, under total anesthesia. Body temperature was maintained using a bed of warm water. An incision of about one cm was made, through which each ovary was highlighted, ligated, and excised. The ligation of the ovaries and the sutures in the abdominal wall were made with resorbable thread, and the skin was sutured with surgical silk. Recovery was quick, with most animals eating food on the same day. The animals partially removed the sutures, the remaining ones being removed on the 4th day after surgery. The healing was complete without complications. Only one animal died during anesthesia before surgical procedures began. The necropsy revealed no apparent changes indicating the cause of death.

#### 2.3.2. NLC Samples Tested for Oral Administration

The tested subjects were divided into ten batches (Table 3). The tested NLC samples—Eveg 1, Eveg 2, Eveg 3, Eveg 4, Evg 5, Eveg 6 (powders)—were dispersed in a slightly sweetened aqueous solution and administered using a gavage, with 10 mL of NLC suspension daily for 14 days. We incorporated into the study the reference dose of 10 mg DSG/kg body weight (b.w.) from the data in the literature, as published in previous studies [29,48].

For batch 5, we administered 0.372 g NLC-Eveg 1/kg body weight, corresponding to 10 mg DSG/kg b.w. and 48.320 mg glycyrrhizic acid; in batch 6, 0.3717 g NLC-Eveg 2 mg/kg b.w, corresponding to 10 mg DSG/ kg body and 48.20 mg glycyrrhizic acid/kg b.w.; for batch 7, 0.3597 g NLC-Eveg 3/kg body weight, corresponding to 10 mg DSG/kg b.w. and 0.294 mg triterpene glycosides/kg b.w. from black cohosh; in batch 8, 0.3597 g NLC-Eveg 4/kg body weight, corresponding to 10 mg DSG/kg b.w. and 29.39 mg triterpene glycosides/kg b.w. from black cohosh as a secondary bioactive compound; for batch 9, 0.323 g NLC-Eveg 5/kg b.w., corresponding to 10 mg DSG/kg body and 3.77 mg polyphenols (3,5,4′-trihidroxi-trans-stilbene); for batch 10, 0.323 g NLC-Eveg 6/kg b.w., corresponding to 10 mg DSG/kg b.w. and 3.77 mg polyphenols (3,5,4′-trihidroxi-trans-stilbene)/kg b.w.

**Figure 4:**
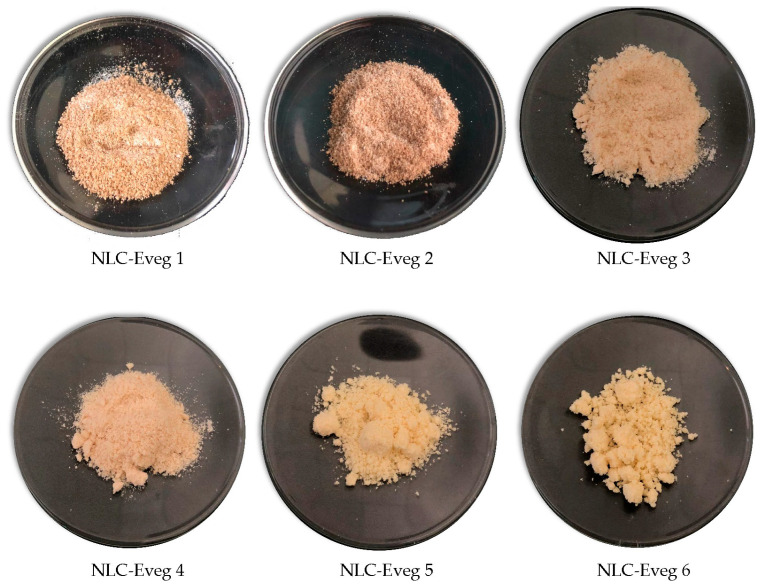
NLC (Eveg 1–6) formulated powders [50] under study.

The control sample prepared as a DSG powder in a non-nano form (10 mg DSG/kg b.w.) was dispersed in an aqueous solution; we used a glucose solution (5%) as a negative control and EG/PG solution (0.1 mg estradiol/kg b.w.) as a positive control [49] (Figure 4).

During the administration period, the animals were observed in terms of their aspect and behavior, fur, appetite, presence of diuresis, and bowel movement; the weight curve was determined by the daily weighing of the animals using an MRC BBa-1200 scale (Premier Farnell, Leeds, England), carried out in the first part of the day after treatment. Food consumption was determined by weighing; water consumption was measured using graded watering containers. The bedding was checked daily, during the weighing of animals, for the presence of water near the watering hole to record water losses.

After day 14, whole blood was collected after sedation and euthanasia in a 15 mL centrifuge tube (TPP—Trasadingen, Switzerland), then allowed for 30 min to clot, and centrifuged at 1000× *g* for 10 min at 8 °C. The resulting supernatant was collected in another tube, constructed at 2000× *g* for 10 min at 8 °C, then moved into 2 mL tubes, and frozen at −80 °C. There were no hemolysed, icteric, or lipemic serum samples. The necropsy looked for macroscopic changes in the organs and their weight, along with measurements of the diameter of the uterus.

#### 2.3.3. Laboratory Tests

The serum samples were subjected to the following biochemistry determinations: (a) markers of bone metabolism—serum alkaline phosphatase, calcium, phosphatemia; (b) markers of carbohydrate metabolism—blood glucose; (c) markers of liver metabolism and hepato-biliary apparatus—transaminases, direct bilirubin, and total bilirubin; (d) markers of lipid profile—triglycerides, HDL-cholesterol, total cholesterol; (e) markers of the functionality of the renal system—creatinine, serum urea, uric acid; (f) markers of inflammation—TNF-alpha, C-reactive protein; (g) other markers—acid phosphatase, total protein; (h) hormonal markers—progesterone and serum estradiol; (i) oxidative stress markers—MDA, GSH.

The laboratory equipment consisted of an automatic open system biochemistry analyzer A25 BioSystems (BIONS Medical Systems, Kerala, India) and a FlexStation multi-mode reader (Molecular Devices, San Jose, CA, USA). The biochemistry reagents were purchased from BioSystems Diagnostics. We performed the biochemistry analyses using the BioSystems multianalyzer and compatible reagents. The markers of oxidative stress (MDA, GSH) and the expression of their proteins TNF-α and 4-HNE were analyzed using the Western blot technique. The protein concentration was determined according to the method described by Bradford [51], using bovine serum albumin (BSA) as the standard.

The degree of lipid peroxidation measured in the form of malondialdehyde (MDA) was determined using the method described by Del Rio et al. [52], using thiobarbituric acid (TBA) as a reactive substance. The fluorescence of the MDA–TBA adducts was quantified (λex/em = 520/549 nm) using a Jasco FP-750 fluorometer (Tokyo, Japan), using Spectra Manager II as software. We calculated the concentration of MDA using a calibration curve with 1,1,3,3-tetramethoxypropane in the range of 0.5–5 μM, and we expressed the results in nM MDA/mg protein.

The determination of the concentration of reduced glutathione (GSH) was carried out using the Glutathione Assay Kit (Sigma-Aldrich, Darmstadt, Germany) based on a kinetic analysis in which catalytic quantities (nmol) of GSH cause a continuous reduction of 5,5′-ditio-bis(2-nitrobenzoic) acid (DTNB) into 5-tio-2-nitrobenzoic acid (TNB); the newly formed GSSH is recycled by glutathione reductase and NADPH.

The optical density of the NTB was measured spectrophotometrically at 412 nm using the FlexStation multi-mode reader. The concentration of GSH in the samples was calculated using a calibration curve made from a stock solution of GSH 10 mM, and the results were expressed in nmol GSH/mg protein.

We used the Western blot technique to analyze the expression of the TNF-α and 4-HNE protein, a method used in detecting specific proteins from a total protein extract, cellular lysate, or serum sample and based on the principle of antigen–antibody reactions. The technique uses electrophoresis in polyacrylamide gel in a denaturing system (SDS-PAGE) to separate proteins according to their molecular weight.

Protein transfer from the migration gel to the PVDF membranes was carried out in a wet system (Bio-Rad, Hercules, CA, USA) at a constant amperage of 350 mA, at 4 °C for 90 min. The protein revelation stage was carried out with the help of the WesternBreeze Chromogenic Kit (Invitrogen, Thermo Fisher Scientific, Waltham, MA USA). After blocking using blocking buffer, the membranes were incubated overnight with a diluted solution (1:250) of primary anti-TNF-α antibodies obtained from goat (Santa Cruz Biotechnology Inc., Heidelberg, Germany) at 4 °C.

The membranes were visualized using the Bio-Rad ChemiDoc Imaging System (Bio-Rad, Hercules, CA, USA). TNF-α protein and 4-HNE expression were quantified using the Bio-Rad Image Lab software program (version 5.2, Bio-Rad, Hercules, CA, USA). The results were expressed as % of the normal control. We used primary polyclonal rabbit antibodies conjugated with bovine serum albumin to detect 4-HNE and rabbit anti-IgG antibodies conjugated with alkaline phosphatase as secondary antibodies.

The statistical analysis was performed with GraphPad Prism v. 7 (GraphPad software, Boston, MA, USA) and the R program (R Foundation for Statistical Computing, Vienna, Austria). We analyzed the sample distributions, normality, and variability. The applied parametric tests were of ANOVA type, and the *p* values related to comparing the groups of animals studied were found using post hoc type tests. The level of statistical significance was 5%.

## 3. Results and Discussions

### 3.1. In Vitro Cell Culture Cytotoxicity Testing: Viability and the Micronucleus Test

#### 3.1.1. Viability Assay (MTT)

**Figure 5:**
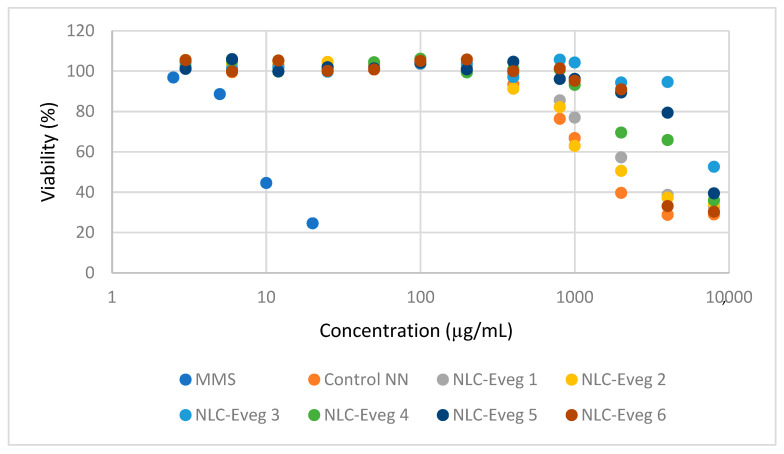
Relative cell viability as a function of test sample concentration (MMS—positive control at concentrations of 5 and 10 μM; Control NN—non-nano diosgenin (DSG)-powder-tested sample; NLC Eveg 1–6 tested samples).

To evaluate the cytotoxicity, we reported the absorbance of treated cultures compared to untreated cultures expressed as a percentage. The lower the viability percentage value, the higher the cytotoxic potential of the test item. If viability falls below 70%, this sample concentration is considered cytotoxic (Figure 5). The cytotoxic effect occurs from 1 mg/mL doses for some compounds (control NN) or even higher amounts for others (NLC-veg 3). Given that the body expects these substances to reach a dose of a maximum of 10 mg/kg, which would correspond in vitro to 10 µg/mL, it can be considered that the therapeutic dose is far from producing cytotoxic effects.

#### 3.1.2. Genotoxicity Test

**Figure 6:**
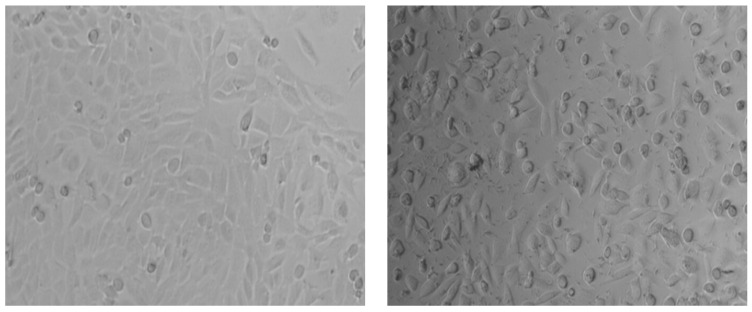
Visualization of cell culture under an inverted microscope, (**left**) untreated, (**right**) incubated with test substances—small black formations are observed among cells.

The cells accepted in the analysis are binucleate cells with clearly delimited cytoplasm, nuclei that do not intertwine, and micronuclei clearly delimited and with the same texture as the main nucleus (Figure 6, Table 4). Turbidity in the environment was observed from the beginning of the administration of the test samples, these being plant extracts that do not dissolve in the culture medium.

The cell culture was not infected with mycoplasma, so the results were not influenced. The cell viability decreases only at very high doses, which may not be toxic per se but may limit the cells’ access to the culture medium. Cytotoxicity is manifested at very high doses, unfeasible in the therapeutic administration of compounds. The micronucleus method did not reveal the genotoxicity of the test compounds, the values obtained being similar to those of the negative control and untreated cell cultures.

### 3.2. In Vivo Tests on a Female Model of Menopausal Rats

#### 3.2.1. Water and Food Consumption

The study subjects ate all food from the feeder without any leftovers observed in the bedding; the water was consumed entirety from drinkers fitted with balls to release the water. These devices are the same as those used throughout the animal’s life, and there should be no reduced water consumption due to their use.

**Figure 7:**
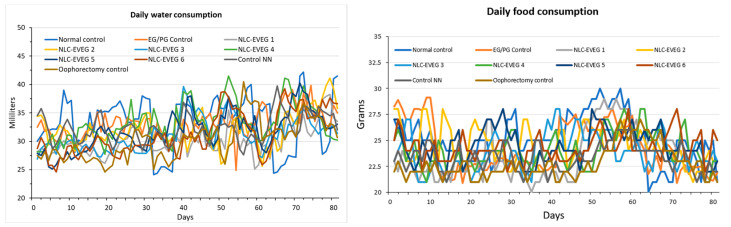
Average consumption of water (1) and feed (2) per batch (Normal control: untreated unoperated control batch; Oophorectomy control: operated, untreated batch; EG/PG Control: operated, treated batch with progesterone/estrogen; Control NN: non-nano diosgenin sample operated, treated batch; NLC Eveg 1–6: tested-sample-treated, operated batches).

Food consumption remained relatively constant, slightly increasing after oophorectomy and decreasing toward the end of the test period. The decrease may be associated with reaching a maximum body weight as seen in the flattening weight curves (Figure 7).

**Figure 8:**
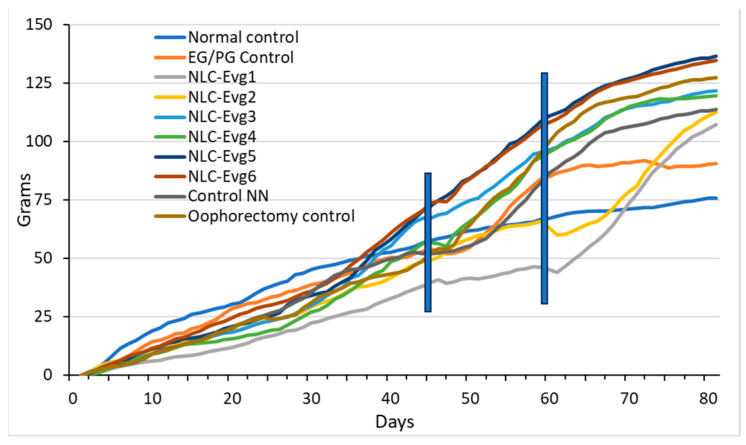
Weight gained by batch. The first vertical line on the left represents the time of oophorectomy, and the second line represents the beginning of the administration of test samples. (Normal control—untreated, unoperated, (normal) control batch; Oophorectomy control—operated, untreated batch; control NN—non-nano-diosgenin-sample-treated batch; NLC-Eveg 1–6—tested-sample-treated batches; EG/PG Control—operated, treated batch with progesterone/estrogen).

There was a constant increase in body weight during the test with some stagnation or decrease in the period immediately after surgery, which is expected because 24 h before surgery, the animals did not receive food, and after surgery, they did not consume food for a short period. In the NLC-Eveg1 and NLC-Eveg2 groups, there was a decrease in body weight in the first days after starting administration, but because the decline is not associated with a reduction in water and food intake or hyperactivity, it may mean an acceleration of internal metabolism. After a short period, these two batches tended to compensate for the deficit (Figure 8).

**Figure 9:**
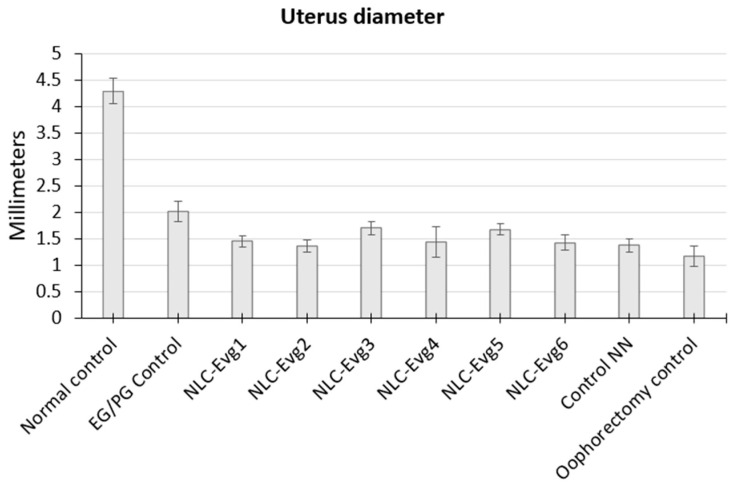
The uterine diameter variated between batches, more prominent in the group that did not receive an oophorectomy and smaller in the group that did not receive treatment.

During the necropsy examination, no toxic changes were found at the level of the examined organs. Since rats have a highly developed sense of smell and taste and had food at their discretion, the animals willingly accepted the compounds to be tested, demonstrating that they tasted and smelled good. No lesions attesting to an irritative action, such as erythema or ulceration, were observed in the digestive tract. Given that the administration took the form of a single dose, it can be considered that each compound is at a maximum concentration from the mouth to the stomach, where it is diluted in its contents. Uterine hypotrophy is visible in the groups where oophorectomy was performed, having a less pronounced manifestation in the group that received synthetic hormones (Figure 9).

All animals were clinically healthy throughout the test; no mortality was found in the test group. Water and food consumption were relatively constant throughout the period. There were no pathologically significant changes in the weight of the underlying morphology of the animals involved in the testing. At the necropsy examination, no lesions of a toxic nature were observed.

#### 3.2.2. Markers of Bone Metabolism (Alkaline Phosphatase, Calcium, Phosphatemia)

**Figure 10:**
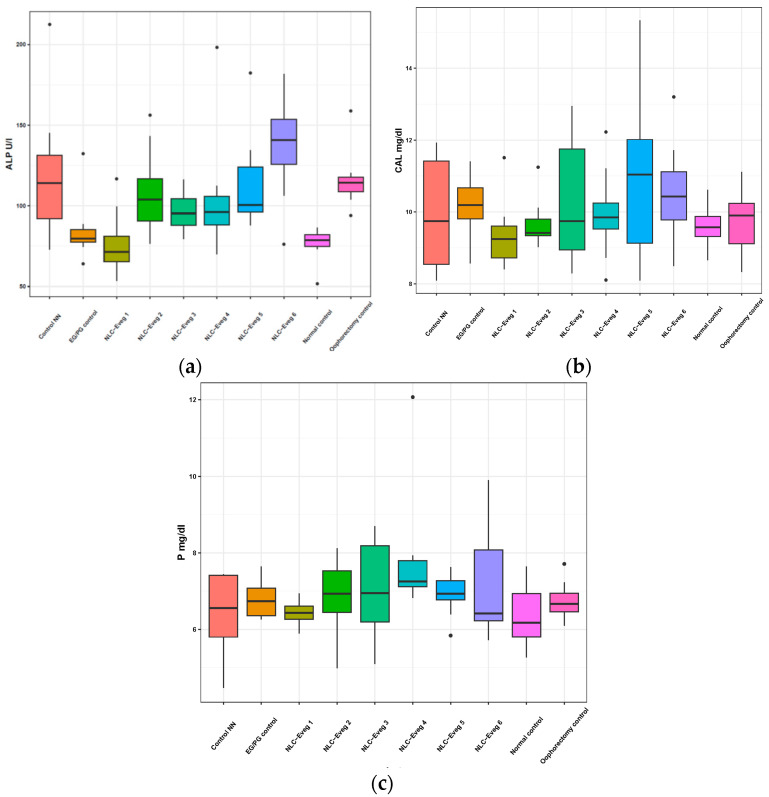
Variation of bone metabolism markers following treatment with nano lipidic pharmaceutical formulations based on diosgenin and resveratrol (NLC-Eveg 1–6). The activity of alkaline phosphatase U/L (**a**), calcemia mg/dL (**b**), phosphatemia mg/dL (**c**); EG/PG control—operated, treated with progesterone/estrogen batch; Control NN—non-nano-diosgenin-sample-treated batch; Normal control—untreated, unoperated control batch; NLC-Eveg 1–6—operated NLC-treated batches.

The results obtained from treatment with the tested NLCs with different compositions of active principles are shown in Figure 10. There were no significant variations in calcemia and phosphatemia between the treated oophorectomy and the normal control groups.

Following the oophorectomy, alkaline phosphatase increased in the oophorectomised groups compared to the group of unoperated animals (*p* < 0.001). In the case of the operated control group, the increase was about 53%. The groups operated and treated with the substance-free NLC (empty NLC, control NN) and NLC-Eveg 1–5 showed elevated values above the normal non-operated control value. Statistically significant increased values were observed in the case of treatment with the NLC-Eveg 6 formula compared with the normal (control) batches (*p* < 0.001), EG/PG control (*p* < 0.01), and NLC-Eveg 1 (*p* < 0.002). In the case of classical hormonal treatment (EG/PG control), the enzymatic activity was similar to the normal control group. Similar values to unoperated animals were also observed in the case of treatment with NLC formulation Eveg 1, which may support the beneficial effect of this treatment.

#### 3.2.3. Marker of Carbohydrate Metabolism—Blood Glucose

**Figure 11:**
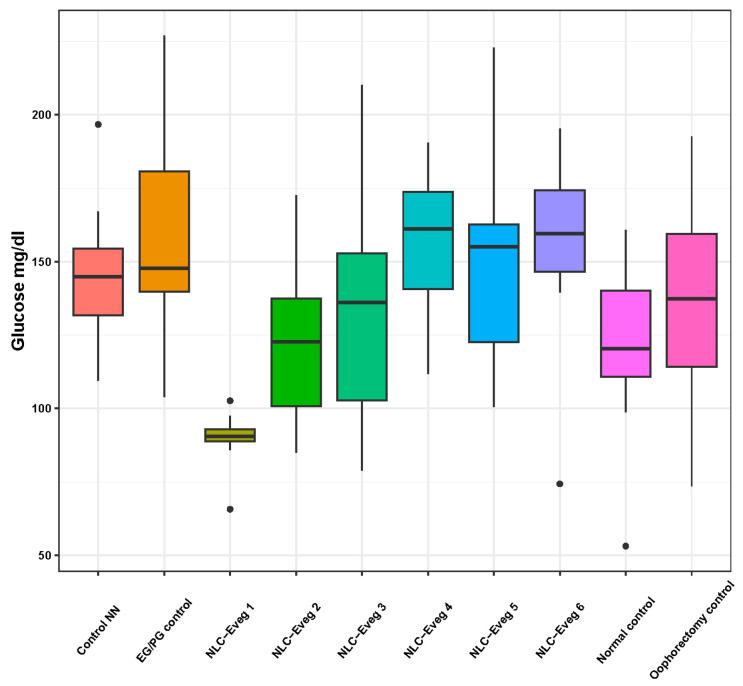
Blood glucose variation following treatment with NLC-Eveg 1–6 (EG/PG control—operated, treated with progesterone/estrogen batch; Control NN—operated non-nano-diosgenin-sample-treated batch; Control normal—untreated, unoperated control batch; Oophorectomy control—operated, untreated batch; NLC-Eveg 1–6—operated NLC treated batches).

Following the determination of the blood glucose levels (Figure 11, Supplementary Table S1), no statistically significant variations existed between the normal control group, the operated control groups, and those treated. In the group treated using NLC-Eveg 1, a significantly lower blood glucose level was observed compared to the group treated with EG/PG (*p* = 0.004) and those treated with NLC-Eveg 4 (*p* = 0.007), NLC-Eveg 5 (*p* = 0.002), and NLC Eveg 6 (*p* = 0.008). Despite the lower blood glucose values in the batch treated using the NLC-Eveg 1 formulation, the results are not significant compared to the normal control group.

#### 3.2.4. Markers of Liver Metabolism and Hepato-Biliary Apparatus—Transaminases, Direct Bilirubin, and Total Bilirubin

We investigated the activity of serum transaminases (aspartate aminotransferase AST and alanin aminotransferases ALT) in the studied groups (control EG/PG—operated, treated with progesterone/estrogen; control NN batch—operated, non-nano-diosgenin-sample-treated batch; control normal—untreated, unoperated control batch; control normal—untreated, operated batch; NLC-Eveg 1–6—operated, NLC-treated batches).

**Figure 12:**
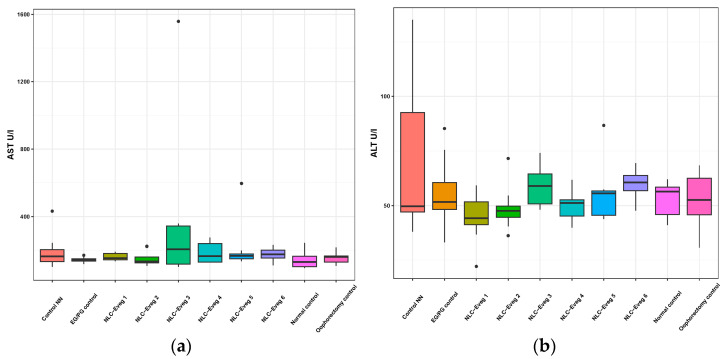
Variation of serum transaminases AST (a) and ALT (**b**) following treatment with NLC-Eveg 1–6.

**Figure 13:**
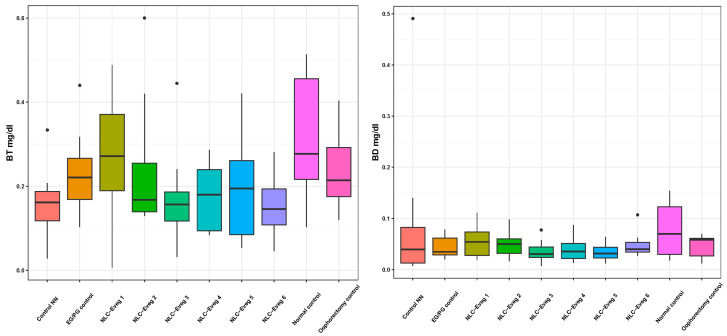
Direct and total bilirubin variation (BD, BT) following treatment with nano lipidic pharmaceutical formulations (NLC-Eveg 1–6) (EG/PG control—operated, treated with progesterone/estrogen batch; Control NN—operated non-nano-diosgenin-sample-treated batch; Normal control—untreated unoperated control batch; Oophorectomy control—operated, untreated batch; NLC-Eveg 1–6—operated NLC-treated batches).

In the case of theNLC-treated and NN and EG/PG control batches, no statistically significant differences were observed compared to the normal control group in any of the markers analyzed (Figure 12 and Figure 13, Supplementary Table S1). The results claim that the pharmaceutical formulations have no side effects on the liver for the period of administration considered in the study.

#### 3.2.5. Markers of Lipid Profile—Triglycerides, HDL Cholesterol, Total Cholesterol

**Figure 14:**
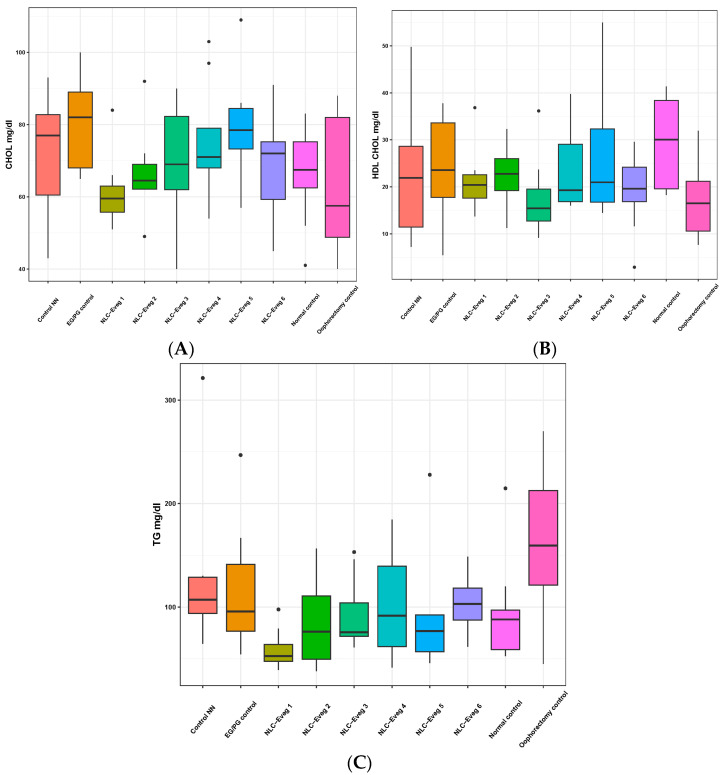
Variation in the lipid profile represented by the markers total cholesterolemia (**A**), serum HDL-cholesterol (**B**), and serum triglycerides (**C**) following treatment with nano lipidic pharmaceutical formulations (NLC-Eveg 1–6).

No statistically significant differences were found in the total cholesterolemia between the groups under study, as presented in Figure 14 (Supplementary Table S1).

The oophorectomy surgery leads over time to a decrease in HDL-cholesterol (−40%) in the operated control group compared to the normal control (*p* = 0.02). Lower levels compared to the normal control group are also observed in batches treated using NCL-Eveg 1, 2, 4, and 5, but the results are not statistically significant compared to the normal control group.

The serum levels of triglycerides increased by 67% in the group of oophorectomized animals compared to the normal control group. Increased values were also recorded in the case of batches treated with EG/PG and NN, but the values are not statistically significant. The lowest triglyceride levels were recorded in the group treated with the Eveg 1 NLC formulation, which also showed lower blood glucose levels, which may support a beneficial therapeutic effect in the case of carbohydrate and lipid metabolism (*p* < 0.01).

#### 3.2.6. Markers of the Renal Function—Serum Creatinine, Serum Urea, Uricemia

**Figure 15:**
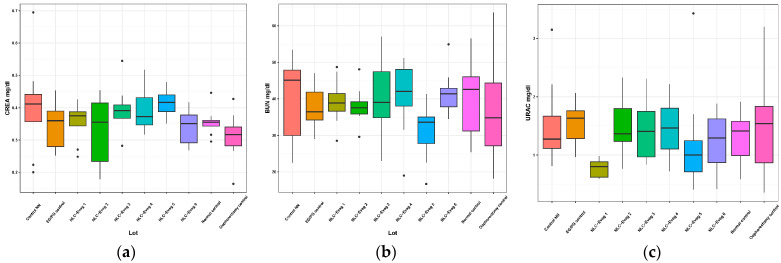
Variation in renal markers: creatinine (**a**), urea (**b**), and uric acid (**c**) following treatment with nano lipidic pharmaceutical formulations (EG/PG control—operated, treated with progesterone/estrogen batch; Control NN—operated non-nano-diosgenin-sample-treated batch; Control normal—untreated, unoperated control batch; Oophorectomy control—operated, untreated batch; NLC-Eveg 1–6—operated NLC-treated batches).

In the operated and treated oophorectomy groups, no statistically significant differences are recorded compared with the normal control group except for the batch treated with NLC Eveg 1, which showed a decrease in uric acid of approximately 40% compared to normal (Figure 15).

#### 3.2.7. Other Markers—Acid Phosphatase and Total Protein

**Figure 16:**
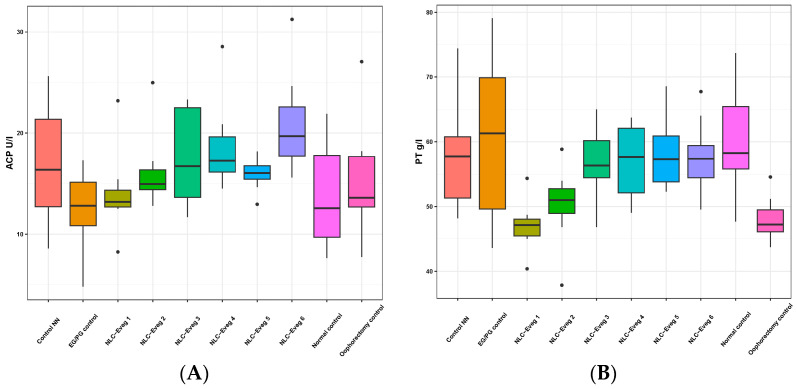
Variation in serum acid phosphatase activity (U/L) (**A**) and total protein (**B**) following treatment with nano lipidic pharmaceutical formulations NLC-Eveg 1–6 (EG/PG control—operated, treated with progesterone/estrogen batch; Control NN—operated non-nano-diosgenin-sample-treated batch; Control normal—untreated, unoperated control batch; Oophorectomy control—operated, untreated batch; NLC-Eveg 1–6—operated NLC-treated batches).

The acid phosphatase activity was not significantly altered by the administered treatments nor in the operated control group compared to the normal control group (Figure 16, Supplementary Table S1). The total serum proteins showed decreases of 20% for the operated control group (*p* = 0.02), 22% for the NLC-Eveg 1 group (*p* < 0.01), and 17% for the NLC-Eveg 2 group. The operated control group showed significant decreases in proteinemia levels compared to the normal control groups and the oophorectomy group treated with standard hormonal treatment, EG/PG (*p* = 0.03). However, the reductions are not of significant diagnostic importance, and several other investigations are required in the context of normal liver function values obtained for all applied treatments (Figure 14).

#### 3.2.8. Hormonal Markers—Progesterone and Serum Estradiol

Progesterone and estradiol are hormonal markers frequently determined against the background of menopause for both diagnostic and monitoring purposes.

**Figure 17:**
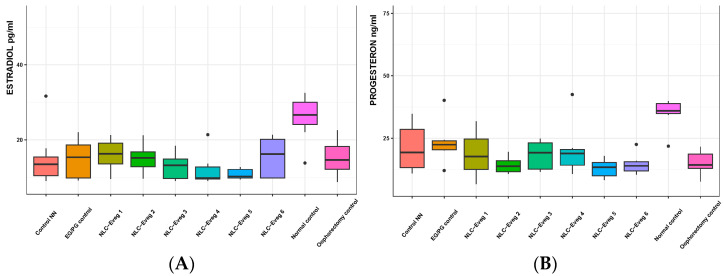
Serum estrogen (**A**) and progesterone (**B**) variation following treatment with nano lipidic pharmaceutical formulations (NLC-Eveg 1–6) (EG/PG control—operated, treated with progesterone/estrogen batch; Control NN—operated non-nano-diosgenin-sample-treated batch; Control normal—untreated, unoperated control batch; Oophorectomy control—operated, untreated batch; NLC-Eveg 1–6—operated, NLC-treated batches).

In the case of oophorectomy, the depletion of these hormones significantly exceeds 50% compared to the normal control group. The obtained results are statistically significant (*p* < 0.01) after comparing the oophorectomy groups with the normal control group, which supports the experimental model applied to experimental animals (Figure 17, Supplementary Table S1).

#### 3.2.9. Markers of Inflammation—TNF-Alpha and C-Reactive Protein

**Figure 18:**
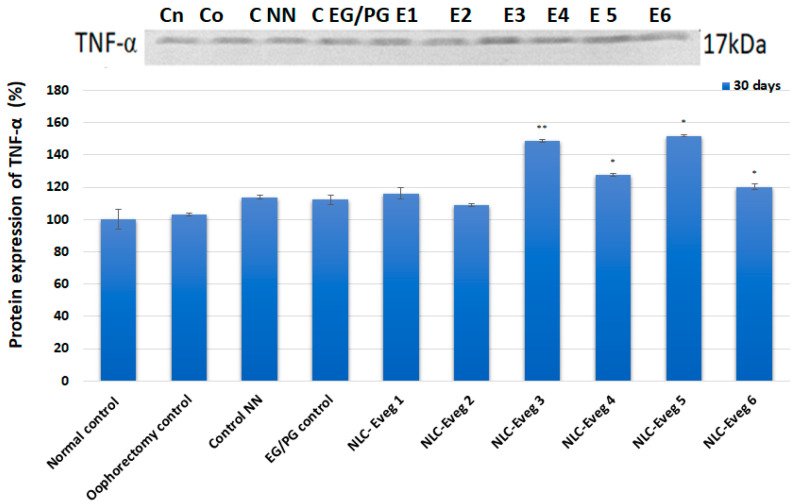
Western blot profile of the TNF-α protein (expressed as % of the normal control) following in vivo treatment after administration of NLC-Eveg 1–6. (control normal—untreated, unoperated batch; control operat—ophorectomy control: operated, untreated batch; control NN—operated, non-nano-diosgenin-sample-treated batch; NLC-Eveg 1–6—operated, NLC-treated batches). Data are calculated as mean value ± SD (*n* = 6) and expressed relative to the corresponding control untreated. * *p* < 0.05; ** *p* < 0.01.

The profile and densitometry of the results obtained in the case of TNF-α protein expression can be observed following the in vivo treatment with different concentrations of pharmaceutical formulations (Figure 18). The TNF-α expression level rises significantly in the Eveg 3, 4, 5, and 6 batches compared to the control batches, with the lowest levels for Eveg 2 and Eveg 1.

The level of C-reactive protein in oophorectomy rats in the experimental model of induced menopause and the normal control group did not show relevant results from the point of view of the diagnosis of inflammation, showing very high variability between animals regardless of the group. In most cases, the C-reactive protein level was below the detection limit, and no values above the reference range were recorded.

#### 3.2.10. The Markers of Oxidative Stress

**Figure 19:**
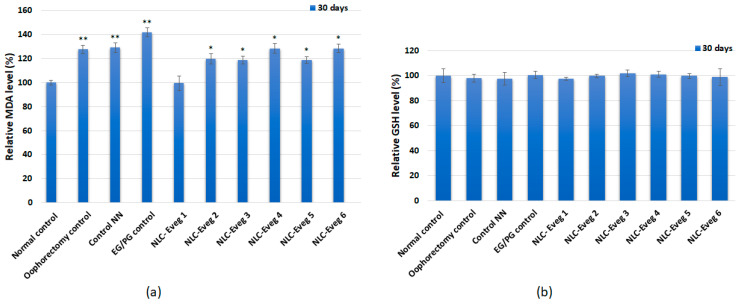
(**a**) The level of malondialdehyde obtained from in vivo treatment after the interval of 30 days after administration of NLC-Eveg 1–6. (**b**) Reduced glutathione concentration obtained from in vivo treatment after 30 days after administration of the pharmaceutical formulations (NLC-Eveg 1–6). Data are calculated as mean value ± SD (*n* = 6) and expressed relative to the corresponding control untreated. * *p* < 0.05; ** *p* < 0.01.

In the case of determining the markers of oxidative stress, the results obtained from the in vivo treatment showed that the lipid peroxidation level increased significantly in the batches treated with the NLC pharmaceutical formulations Eveg 2, 3, 4, 5, and 6, respectively. In contrast, in the case of the batch treated with Eveg 1, the MDA level was maintained at the level of the untreated control (Figure 19). The low glutathione concentration (GSH) showed no significant changes in the batches tested compared to the control group (Figure 17).

**Figure 20:**
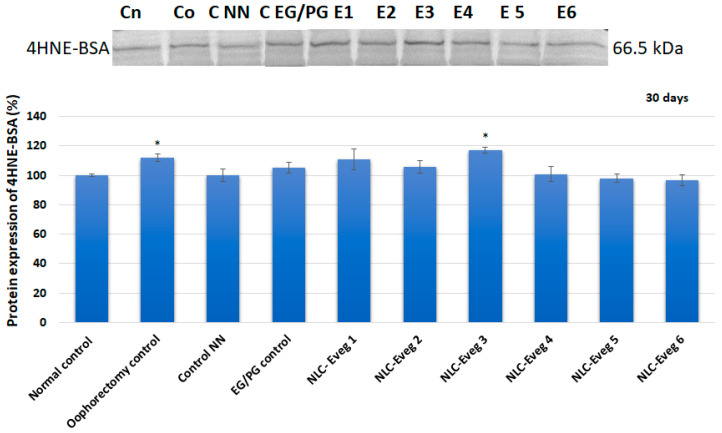
Western blot profile of 4HNE-BSA following in vivo treatment after 30 days of administration of pharmaceutical formulations (NLC Eveg 1–6) (control normal—untreated, unoperated control batch; control operat—oophorectomy control: operated, untreated batch; control NN—operated non-nano-diosgenin-sample-treated batch; NLC-Eveg 1–6—operated NLC-treated batches). Data are calculated as mean value ± SD (*n* = 6) and expressed relative to the corresponding control untreated. * *p* < 0.05.

The level of 4-hydroxynonenal increases significantly in the untreated oophorectomized batch and NLC-Eveg-3-treated batch, while in the other batches, there are no significant changes compared to the untreated batch (Figure 20).

## 4. Discussion

The major findings of our study are that (a) diosgenin (Dioscorea villosa L. standardized extract) formulated in various lipidic nanocapsules (NLCs) in combination with glycyrrhizic acid (Glycyrrhiza glabra standardized extract, Eveg 1 and Eveg 2), TTG (triterpene glycosides) (Actaea racemosa standardized extract, Eveg 3 and Eveg 4), and resveratrol polyphenols (Polygonum cuspidatum standardized extract, Eveg 5 and Eveg 6) tested on cultured human cells using the micronucleus method did not reveal the genotoxicity of the test compounds, the values obtained being similar to those of negative control and untreated cell cultures; (b) the NLC formulas tested (Eveg 1–6) administered orally to surgically induced menopause female rats model showed no influence on the bone metabolism, carbohydrate metabolism, liver and biliary markers, lipid blood profile, acid phosphatase, or inflammatory markers; (c) the NLC formulas tested (Eveg 1–6) in the menopause model also did not influence the markers of oxidative stress or the hormonal markers.

The toxicity of oral nanoparticles can occur topically, when in contact with intestinal cells, or systemically after they are translocated into the bloodstream. Due to the increased exposure of nanoparticles to the environment, there is a need for a better assessment of nanotoxicity and the imposition of methods for the more efficient use of nanoparticles and appropriate disposal methods to help reduce adverse effects [53]. A detailed understanding of the mechanisms responsible for the toxicity of nanoparticles and their interaction with the environment is required, along with factors contributing to their toxic effects, with specific information about metal nanoparticles such as gold, silver, superparamagnetic iron oxide, platinum nanoparticles, and zinc; in this way, By thoroughly examining their negative effects on human cell lines and analyzing clinical data from various in vitro and in vivo models, we can better understand the toxic consequences and safety profiles of nano-sized particles [54].

Toxicity studies are often neglected, as most materials used to produce nanoparticles are biocompatible and biodegradable [55]. Often, toxicity is associated only with materials that are part of nanoparticles, but it is well known that the pharmacokinetic property of a drug or excipient can change considerably when incorporated into a nanoparticulate system [56,57].

The oral toxicity of nanoparticles can occur topically, when in contact with intestinal cells, or systemically after they are translocated into the bloodstream. (a) Local toxicity can be caused by the direct interaction of nanoparticles with intestinal cells, which can influence different properties of nanoparticles such as size and charge [58]. (b) In the case of systemic toxicity, all nanoparticle characteristics that affect both their translocation and interaction with different tissues shall be considered [59]. To develop modified-release nano pharmaceutical forms and dietary supplements, performing the toxicity testing step is paramount, run on in vitro cultured plant, animal, and human cells (cell cultures) and then on a limited number of laboratory animals [60].

A micronucleus is the third irregular nucleus that forms during anaphase, mitosis, or meiosis [61,62]; more than one micronucleus will form when more genetic damage has occurred [63]. In our study, the micronucleus method did not reveal the genotoxicity of the test compounds, the values obtained being similar to those of the negative control and untreated cell cultures.

### Influence of the Tested NLCs on The Specific Biochemical Markers

The study aimed to achieve a pharmaco-toxicological profile and to evaluate the effects that treatment with different pharmaceutical formulations based on lipid nanoparticles with diosgenin and resveratrol had on the body in an experimental model of menopause induced by oophorectomy in female Wistar rats.

Menopause is often accompanied by imbalances in this metabolism, leading over time to an increase in bone turnover; with the onset of osteoporosis, menopause decreases bone-forming osteoblasts along with osteoblastic lineage cells such as osteocytes [64,65]. Alkaline phosphatase (Palc), calcemia (Ca), and phosphatemia (Ph) were tested as bone metabolism markers. The increased alkaline phosphatase activity correlates with imbalances in the osteoblastic/osteoclastic metabolic processes, resulting in bone formation and remodeling. In the case of osteoporosis, increases in serum alkaline phosphatase are often observed, but variations in calcium or phosphatemia are not always relevant to diagnosis, the determinations helping initiate an optimal treatment.

In the present study, following an oophorectomy, the alkaline phosphatase increased in the oophorectomized groups compared to the group of unoperated animals (*p* < 0.001). The levels of alkaline phosphatase displayed the following patterns: (a) they were comparable between the group treated with estrogen/progesterone and the unoperated group; (b) there was an elevation in the group that underwent surgery but did not receive treatment (control group), in the non-loaded NLC (Control NN) group, and in the NLC-Eveg 2–6 groups, with the highest level observed in the NLC 6 group. This increase is likely attributed to the active biomolecules diosgenin and resveratrol polyphenols contained within Sylibum marianum oil. However, this elevation was not statistically significant, as indicated in Supplementary Table S1. Moreover, the Eveg-1 formula of diosgenin and glycyrrhizic acid nano-encapsulated in Oenothera biennis oil showed a level comparable to the unoperated and pharmaceutically treated groups, suggesting a protective effect against menopause-induced bone catabolism of Eveg-1.

Previous studies showed that in a fenugreek-supplemented diet given to diabetic obese KK-Ay mice, DSG was shown to promote adipocyte differentiation, potentially ameliorating the glucose metabolic disorder associated with obesity [66]. In the present study, no statistically significant variations existed between the normal control group, the operated control groups, and those treated. In the group treated with NLC-Eveg-1, a significantly lower blood glucose level was observed compared to the EG/PG-treated group; although a potential glucose-lowering effect might appear using the tested formula containing soy oil and DSG, it has no statistical significance. The previous study on a diabetic rat model showed an insignificant reduction in blood sugar levels compared to the control, but with potential benefits in hyperglycemic individuals [67,68].

The most commonly determined markers for identifying malfunctions in the liver tissue are (1) the activity of serum transaminases (aspartate aminotransferase AST and alanine aminotransferases ALT) as markers of liver damage, and (2) the direct and total serum bilirubin as markers of bile transport. Diosgenin was previously studied for its hepatoprotective effects, especially in chemical-induced liver injury, via oral administration of Trigonella whole seed powder (5% in the diet) for 21 days in alloxan-induced diabetic Wistar rats. A study found it helps to ameliorate liver damage, reduce elevated levels of liver enzymes [69], and protect against oxidative-stress-induced damage, which is relevant to liver health. In 2012, Liu Kang et al. showed that DSG can ameliorate the negative impacts of palmitate-induced endothelial disruptions and insulin resistance via blocking the IKKβ and IRS-1 pathways and inhibiting TNF-α and IL-6 production in the endothelial cells [70]. While primarily focused on endothelial function, the results give insight into diosgenin’s broader metabolic and protective roles applicable to liver metabolism.

The total serum cholesterol, HDL cholesterol, and serum triglycerides are standard markers for the risk of atherosclerosis, generally correlated with cardiovascular side effects. Serum triglycerides are most often correlated with the blood glucose values.

In the present study, the ablation of the gonadal apparatus in the surgical menopause model generated a decrease in HDL-cholesterol (−40%) in the operated control group compared to the normal control (*p* = 0.02) and in the EG/PG-treated group, which corresponds to clinical observations that have shown that increased cardiovascular risk with the development over time of atherosclerosis most often accompanies menopause and hormone replacement therapy. In the case of Eveg1 treatment (−2.57%), the cholesterol values were lower than the normal unoperated controls but the decrease was not statistically significant. The serum levels of triglycerides increased by 67% in the group of oophorectomized untreated animals compared to the normal (unoperated, untreated) control group. The triglyceride levels in the EG/PG group were lower compared to the menopause (untreated) model group (−27.46%), but also in the NLC-Eveg-1–6-treated groups, with the lowest TG levels being recorded in the NLC-Eveg-1-treated group (−63.36%), containing DSG and glycyrrhizic acid nanoencapsulation in evening primrose (Oenothera biennis) oil, which also showed lower blood glucose levels (−34.66%), and may support a beneficial therapeutic effect on the carbohydrate and lipid metabolism (*p* < 0.01).

Creatinine and serum urea are standard parameters for determining glomerular filtration dysfunctions as they occur in various kidney diseases. Uric acid is a marker of purinic metabolism that the renal route eliminates, associated with increased creatininemia and serum urea in kidney diseases.

Jin et al. showed in 2020 that diosgenin had renoprotective properties on aristolochic acid I (AA-I) nephropathy (AAN) via intragastric treatment with 30 mg/kg/d diosgenin two hours before exposure to 10 mg/kg/d AA-I for four consecutive weeks by inhibiting the AIF expression and the cleaved form of Caspase-3, thereby alleviating the apoptosis triggered by AA-I, by increasing the expression of mitochondrial-dynamic-related proteins including DRP1 and MFN2. Diosgenin inhibited AA-I-evoked autophagy via ULK1-mediated inhibition of the mTOR pathway and potentially exerted a protective effect against AA-I-induced renal damage [71].

In our study, no significant changes were recorded in the renal function (CREA, BUN), showing a renal safety profile in all the tested formulas; no statistically significant differences were recorded in the other tested formulas (Eveg 2–6) compared with the normal control group in the uric acid plasma levels. However, the batch treated with NLC-Eveg 1 showed a significant decrease in uric acid of approximately 40% compared to the normal group, with a further potential to further support a healthy metabolism in the protein and renal milieu, as supported by previous research [72].

The inflammation markers (TNF alpha and CRP) did not show relevant results from the point of view of the diagnosis of inflammation, showing very high variability between animals regardless of the group, and no values above the reference range were recorded.

The lipid peroxidation level increased significantly in the batches treated with the NLC pharmaceutical formulations Eveg 2 –6. However, in the Eveg 1 treated group, the MDA level was at the same level as the untreated control, suggesting an anti-inflammatory antioxidant role of the active principles in Eveg 1, i.e., diosgenin and glycyrrhizic acid encapsulated in evening primrose oil; glutathione concentration (GSH) showed no significant changes among the batches under study.

Overall, following surgically induced menopause, the biochemical parameters revealing (a) bone metabolism—serum alkaline phosphatase, calcium, phosphatemia; (b) carbohydrate metabolism—blood glucose; (c) liver metabolism and hepato-biliary apparatus—transaminases, direct bilirubin and total bilirubin; (d) lipid profile—triglycerides, HDL-cholesterol, total cholesterol; (e) the renal system—creatinine, serum urea, uric acid; (f) inflammation—TNF-alpha, C-reactive protein; (g) acid phosphatase, total protein; (h) hormonal markers—progesterone and serum estradiol; were significantly altered. Pharmacological intervention did not statistically change the biochemical status compared to the operated untreated subjects, showing a good safety profile. Notably, among the six NLCs under study, NLC-Eveg 1, formulated with active DSG and licorice extract encapsulated in evening primrose oil, showed the most beneficial effects in maintaining the biochemical parameters close to the unoperated control batch, different from Eveg 2, which contained the same active principles but encapsulated in soy oil.

Evening primrose oil (EPO), derived from the seeds of the Oenothera biennis wildflower, is notably rich in gamma-linolenic acid (GLA), an omega-6 essential fatty acid. This composition is unique to evening primrose oil, compared to the other oils in the nano lipid matrix formulations used in the study (Silibum marianum oil, soy oil, and flaxseed oil) [73]. The high concentration of GLA in evening primrose oil, typically ranging from 8 to 10%, is significant due to its role as a precursor of anti-inflammatory eicosanoids via the production of prostaglandins series 1 and leukotrienes series 3 [74], essential for the proper functioning of human tissues; in our formula, it may contribute to the therapeutic efficacy observed for NLC-Eveg 1.The presence of linoleic acid (LA), which constitutes about 70–74% of EPO, further enhances its biological activity [75]. While diosgenin and glycyrrhizic acid have their own recognized pharmacological activities, the distinctive results of NLC-Eveg 1 suggest a synergistic or predominant role of evening primrose oil in the formulation. This aligns with the understanding that omega-6 fatty acids, particularly GLA, have multifaceted roles in the body, including anti-inflammatory, antioxidant, and pain-relieving effects [76]. Future studies should focus on isolating the effects of evening primrose oil from those of diosgenin and glycyrrhizic acid as comparative studies using formulations with and without evening primrose oil while keeping the other components constant.

## 5. Conclusions

The present study investigated the safety and pharmacotoxicology of six nanoforms for oral administration targeted to support menopausal dysfunctions, as part of broader research, using (a) a genotoxicity study of the tested formulas using the micronucleus method; (b) a surgically induced menopause model in female Wistar laboratory rodents. The micronucleus test did not reveal the genotoxicity of the tested compounds; the menopause model showed no significant safety concerns for the six tested formulas evaluated using blood biochemical parameters on the liver function, renal function, lipid and carbohydrate metabolism, inflammatory markers, bone metabolism, markers of oxidative stress, and hormonal markers. Moreover, the plasma levels showed potential the hypoglycemic, hypolipidemic, hypouricemic, and antioxidant potential of a tested formula containing nano diosgenin and glycyrrhizic acid encapsulated in evening primrose lipid phase. Further studies on metabolic disease models could be performed on the selected formula.

## 6. Limitations

The present study has limitations. The induced menopause model was surgically performed, which can be considered a surgical trauma compared to physiological menopause. Another limitation is the treatment duration of 30 days, both for the tested formulas and for the positive control treated with the conventional hormone replacement combination.

## Figures and Tables

**Table 1:** Qualitative NLC chemical composition.

NLC Sample Qualitative Composition	Pharmaceutical Formula Function
Cetyl palmitate, glyceryl monostearate	Lipid mixture (fatty phase)
Liquid oil phase: (EPO/SBO/SMO/FSO)	
Cocoa butter, Tween 20, Span 80, Poloxamer 188, Phosphatidylcholine	Surfactants and Co-surfactants (aqueous phase)
Wild yam extract and licorice extract or black cohosh extract or resveratrol	Standardized plant extracts

**Table 2:** Composition of the NLC samples taken for study: encapsulated active principles and liquid oil phase.

No.	Sample Code *	Encapsulated Active Principles	Liquid Oil Phase
1	NLC-Eveg 1	DSG	EPO
		GA	
2	NLC-Eveg 2	DSG	SBO
		GA	
3	NLC-Eveg 3	DSG	SMO
		TTG	
4	NLC-Eveg 4	DSG	FSO
		TTG	
5	NLC-Eveg 5	DSG	FSO
		PP	
6	NLC-Eveg 6	DSG	SMO
		PP	

* NLC—nanostructured lipid carrier; EPO—evening primrose oil, SBO—soybean oil; FSO—flaxseed oil, SMO—Silybum marianum oil, DSG—diosgenin (wild yam standardized extract), GA—glycyrrhizic acid (licorice standardized extract), TTG—triterpene glycosides (black cohosh standardized extract); PP—polyphenols (resveratrol).

**Table 3:** The tested animals were distributed in 10 batches as follows.

Batch No.	Batch Description	Description of the Batch
1	Normal control	Healthy, untreated, unoperated rat females;
2	Oophorectomy control	Oophorectomised rat females without treatment;
3	EG/PG control	Oophorectomised female rats treated with progesterone/estrogen according to standard treatment in menopause;
4	Control NN	Oophorectomised female rats treated with unloaded NLC pharmaceutical formula (with DSG active substances);
5	NLC-Eveg 1	Female oophorectomised rats treated with lipid nanoparticles with an active substance corresponding to NLC formula 1;
6	NLC-Eveg 2	Oophorectomised female rats treated with lipid nanoparticles with active substance corresponding to NLC formula 2;
7	NLC-Eveg 3	Oophorectomised female rats treated with lipid nanoparticles with active substance corresponding to pharmaceutical formula 3;
8	NLC-Eveg 4	Oophorectomised female rats treated with lipid nanoparticles with active substance corresponding to pharmaceutical formula 4;
9	NLC-Eveg 5	Oophorectomised female rats treated with lipid nanoparticles with active substance corresponding to pharmaceutical formula 5;
10	NLC-Eveg 6	Oophorectomised female rats treated with lipid nanoparticles with active substance corresponding to pharmaceutical formula 6.

**Table 4:** Percentage of micronuclei in binucleate cells at exposure time 4 h.

Compound/Exposure Time	4 h		24 h	
	Binucleated Cells (%)	Micronuclei (%)	Binucleated Cells (%)	Micronuclei (%)
Control (untreated)	72.1	2.2	68.1	1.8
Control (DMSO 0.1%)	75.0	2.4	67.8	2.3
Control NN	77.0	1.7	72.3	1.9
NLC-Eveg 1	68.8	2.3	69.3	2.3
NLC-Eveg 2	66.2	2.5	66.2	2.9
NLC-Eveg 3	71.0	1.9	69.9	2.2
NLC-Eveg 4	63.3	1.6	70.3	3.2
NLC-Eveg 5	71.1	2.5	66.9	3.0
NLC-Eveg 6	68.0	2.2	75.5	2.6
C positive (MMS 5 µg/mL) 4 h/24 h	52.3	17.3	50.8	18.6
C positive (MMS 10 µg/mL) 4 h/24 h	48.6	19.1	49.3	22.6

## Data Availability

Data is contained within the article or supplementary material.

## Supplementary Materials

The following supporting information can be downloaded at https://www.mdpi.com/article/10.3390/nu15234951/s1: Table S1: Statistical data on biochemistry parameters.
