# Supplementary material for: Safety of Innovative Nanotechnology Oral Formulations Loaded with Bioactive Menopause Molecules: Influence of Genotoxicity and Biochemical Parameters on a Menopausal Rat Model
Source: Nutrients. 2023 Nov 29;15(23):4951. doi: 10.3390/nu15234951 (PMC10708031; doi:10.3390/nu15234951)
Supplement: Supplementary file 1 [file nutrients-15-04951-s001.zip › nutrients-2702906-supplementary.pdf]

Table S1. Biochemical parameters on blood samples in surgically induced menopausal model in female Wistar rats

Table no.1 Serum alkaline phosphatase (U./L) level in oophorectomized rat females in an experimental model of induced menopause

	N	Mean	Std. Dev.	Min	Item 25	Paragraph 75	Max
**Normal control**	8	76.234	10.864	51.72	74.732	82.188	86.48
**Operated control**	8	116.59	18.971	93.99	108.825	117.645	158.8
**Control EG/PG**	8	85.188	20.354	64.07	77.552	85.285	132.32
**Control NN**	8	120.628	43.87	72.83	92.073	131.27	212.42
**NLC Eveg 1**	8	76.98	20.92	53.45	65.472	81.16	116.72
**NLC Eveg 2**	8	108.269	28.187	76.45	90.602	116.79	156.19
**NLC Eveg 3**	8	96.589	11.872	79.49	87.998	104.362	116.27
**NLC Eveg 4**	8	106.539	39.131	69.92	88.068	105.872	198.19
**NLC Eveg 5**	8	114.591	31.401	87.92	96.25	124.045	182.33
**NLC Eveg 6**	8	137.601	34.633	76.19	125.805	153.485	181.73
**ANOVA P<0.000321 *****							

Table no.2 Level of calcium (mg/dL) in oophorectomised rat females in an experimental model of induced menopause

	N	Mean	Std. Dev.	Min	Item 25	Paragraph 75	Max
**Normal control**	8	9.605	0.585	8.656	9.316	9.874	10.619
**Operated control**	8	9.749	1.01	8.331	9.118	10.244	11.112
**Control EG/PG**	8	10.157	0.901	8.567	9.812	10.671	11.409
**Control NN**	8	9.915	1.633	8.093	8.543	11.414	11.927
**NLC Eveg 1**	8	9.396	0.987	8.406	8.719	9.612	11.511
**NLC Eveg 2**	8	9.689	0.71	9.024	9.339	9.804	11.246
**NLC Eveg 3**	8	10.257	1.735	8.291	8.946	11.751	12.95
**NLC Eveg 4**	8	9.961	1.294	8.106	9.523	10.247	12.225
**NLC Eveg 5**	8	10.993	2.382	8.096	9.132	12.016	15.328
**NLC Eveg 6**	8	10.493	1.549	8.496	9.777	11.12	13.199
**ANOVA P=0.502**							

Table no.3 Level of phosphathema (mg/dL) in females of oophoroctomised rats in experimental model of induced menopause

	N	Mean	Std. Dev.	Min	Item 25	Paragraph 75	Max
**Normal control**	8	6.381	0.862	5.27	5.808	6.935	7.64
**Operated control**	8	6.763	0.507	6.1	6.458	6.945	7.71
**Control EG/PG**	8	6.776	0.48	6.26	6.36	7.075	7.64
**Control NN**	8	6.424	1.046	4.48	5.8	7.41	7.44
**NLC Eveg 1**	8	6.416	0.331	5.89	6.265	6.607	6.94

**NLC Eveg 2**	8	6.829	1.125	4.99	6.447	7.528	8.12
**NLC Eveg 3**	8	7.046	1.255	5.1	6.193	8.183	8.7
**NLC Eveg 4**	8	7.889	1.734	6.82	7.115	7.795	12.07
**NLC Eveg 5**	8	6.924	0.594	5.84	6.773	7.275	7.63
**NLC Eveg 6**	8	7.12	1.461	5.72	6.225	8.075	9.9
**ANOVA P=0.171**							

Table no. 4 Blood glucose level (mg/dL) in oophoroctomized rats in experimental model of induced menopause

	N	Mean (mg/dL)	Std. Dev.	Min (mg/dL)	Item 25	Paragraph 75	Max (mg/dL)
Normal control	8	119.748	33.87	53.12	110.807	140.075	160.86
Operated control	8	136.589	39.779	73.48	114.18	159.395	192.64
Control EG/PG	8	158.333	38.256	103.87	139.765	180.642	226.95
Control NN	8	145.801	27.662	109.4	131.812	154.46	196.69
NLC Eveg 1	8	89.246	10.827	65.7	88.758	92.968	102.63
NLC Eveg 2	8	121.721	29.689	85	100.865	137.402	172.58
NLC Eveg 3	8	132.974	42.205	78.92	102.745	152.882	210.12
NLC Eveg 4	8	155.572	28.294	111.72	140.715	173.7	190.45
NLC Eveg 5	8	149.512	39.229	100.49	122.687	162.72	222.76
NLC Eveg 6	8	154.637	37.729	74.34	146.588	174.372	195.24
ANOVA P<0.00253 **							

Table no. 5 Level of AST (U/L) activity in oophorectomized rat females in an experimental model of induced menopause

	N	Mean	Std. Dev.	Min	Item 25	Paragraph 75	Max
Normal control	8	141.974	50.564	94.75	103.498	164.132	243.19
Operated control	8	155.122	34.074	108.96	130.62	167.047	217.23
Control EG/PG	8	143.345	18.514	118.49	136.237	149.468	170.66
Control NN	8	193.171	106.884	102.99	133.04	204.19	432.06
NLC Eveg 1	8	160.655	21.949	135.2	144.065	181.01	191.4
NLC Eveg 2	8	151.166	45.898	107.96	125.43	160.64	224.22
NLC Eveg 3	8	374.861	489.758	101.8	118.465	343.335	1558
NLC Eveg 4	8	186.839	63.478	127.56	130.865	240.45	275.04
NLC Eveg 5	8	215.806	155.063	135.75	149.353	177.815	596.36
NLC Eveg 6	8	173.108	40.871	110.67	154.165	200.658	231.24
ANOVA P=0.235							

Table no. 6 ALT (U/L) activity level in oophorectomized rats in an experimental model of induced menopause

	N	Mean	Std. Dev.	Min	Item 25	Paragraph 75	Max

Normal control	8	53.043	8.226	41.06	45.853	58.485	61.99
Operated control	8	52.53	12.607	30.67	45.803	62.51	68.38
Control EG/PG	8	55.802	16.825	33.16	48.17	60.53	85.3
Control NN	8	69.026	34.223	38	47.02	92.572	135.1
NLC Eveg 1	8	44.17	11.304	22.11	41.272	51.66	59.16
NLC Eveg 2	8	48.992	10.642	36.2	44.578	49.752	71.58
NLC Eveg 3	8	58.906	9.334	48.13	50.75	64.532	74.06
NLC Eveg 4	8	49.905	6.687	39.91	45.172	52.612	61.78
NLC Eveg 5	8	55.811	13.711	43.9	45.575	56.682	86.72
NLC Eveg 6	8	59.305	7.421	47.65	56.772	63.828	69.38
ANOVA P=0.124							

Table no. 7 BD level in oophorectomy rat females in an experimental model of induced menopause

	**N**	**Mean**	**Std. Dev.**	**Min**	**Item 25**	**Paragraph 75**	**Max**
Normal control	8	0.078	0.054	0.018	0.03	0.123	0.154
Operated control	8	0.046	0.023	0.012	0.027	0.061	0.07
Control EG/PG	8	0.044	0.021	0.02	0.029	0.062	0.078
Control NN	8	0.101	0.164	0.007	0.013	0.082	0.491
NLC Eveg 1	8	0.055	0.032	0.019	0.028	0.073	0.111
NLC Eveg 2	8	0.05	0.026	0.016	0.032	0.06	0.098
NLC Eveg 3	8	0.036	0.022	0.007	0.024	0.044	0.077
NLC Eveg 4	8	0.04	0.025	0.013	0.022	0.051	0.087
NLC Eveg 5	8	0.035	0.019	0.012	0.023	0.044	0.064
NLC Eveg 6	8	0.049	0.026	0.027	0.034	0.053	0.107
ANOVA P= 0.469							

Table no. 8 BT level in oophorectomy rat females in experimental model of induced menopause

	**N**	**Mean**	**Std. Dev.**	**Min**	**Item 25**	**Paragraph 75**	**Max**
Normal control	8	0.311	0.154	0.103	0.216	0.456	0.514
Operated control	8	0.243	0.101	0.12	0.175	0.292	0.403
Control EG/PG	8	0.231	0.111	0.103	0.169	0.267	0.44
Control NN	8	0.161	0.09	0.029	0.118	0.188	0.334
NLC Eveg 1	8	0.268	0.15	0.006	0.189	0.371	0.488
NLC Eveg 2	8	0.245	0.172	0.129	0.139	0.255	0.6
NLC Eveg 3	8	0.172	0.13	0.032	0.117	0.186	0.445
NLC Eveg 4	8	0.178	0.084	0.084	0.094	0.24	0.286
NLC Eveg 5	8	0.196	0.127	0.053	0.085	0.261	0.421
NLC Eveg 6	8	0.155	0.082	0.045	0.108	0.194	0.281

ANOVA P= 0.21

Table no. 9 Total cholesterolemia level (mg/dL) in oophorectomized rats in an experimental model of induced menopause

	N	Mean	Std. Dev.	Min	Item 25	Paragraph 75	Max
**Normal control**	8	66.5	14.233	41	62.5	75.25	83
**Operated control**	8	63	18.959	40	48.75	82	88
**Control EG/PG**	8	81.125	13.517	65	68	89	100
**Control NN**	8	72.125	16.444	43	60.5	82.75	93
**NLC Eveg 1**	8	61.375	10.419	51	55.75	63	84
**NLC Eveg 2**	8	66.775	12.163	49	62.15	69	92
**NLC Eveg 3**	8	69.375	17.345	40	62	82.25	90
**NLC Eveg 4**	8	75.625	16.248	54	68	79	103
**NLC Eveg 5**	8	79.5	15.203	57	73.25	84.5	109
**NLC Eveg 6**	8	68.5	14.081	45	59.25	75.25	91
**ANOVA P= 0.145**							

Table no. 10 Level of HDL-cholesterolemia (mg/dL) in females of oophoroctomized rats in the experimental model of induced menopause

	N	Mean	Std. Dev.	Min	Item 25	Paragraph 75	Max
**Normal control**	8	29.644	9.99	18.27	19.6	38.35	41.34
**Operated control**	8	17.747	9.159	7.67	10.587	21.18	31.9
**Control EG/PG**	8	24.005	11.044	5.5	17.765	33.608	37.76
**Control NN**	8	23.972	15.665	7.28	11.478	28.625	49.74
**NLC Eveg 1**	8	21.364	7.083	13.72	17.598	22.6	36.84
**NLC Eveg 2**	8	22.69	6.922	11.29	19.2	25.988	32.28
**NLC Eveg 3**	8	17.869	8.574	9.16	12.758	19.535	36.15
**NLC Eveg 4**	8	23.852	9.435	16.04	16.843	29.07	39.74
**NLC Eveg 5**	8	26.62	13.914	14.5	16.753	32.3	54.93
**NLC Eveg 6**	8	18.98	8.465	2.92	16.858	24.218	29.56
**ANOVA P= 0.383**							

Table no. 11 Triglyceremia level (mg/dL) in females of oophorectomized rats in an experimental model of induced menopause

	N	Mean	Std. Dev.	Min	Item 25	Paragraph 75	Max
**Normal control**	8	96.028	53.029	52.8	59.025	97.005	214.75
**Operated control**	8	160.496	72.602	45.1	121.202	212.61	269.92
**Control EG/PG**	8	116.42	64.793	54.49	76.698	141.228	246.95

**Control NN**	8	128.622	81.681	64.6	93.935	128.93	321.51
**NLC Eveg 1**	8	58.803	20.093	39.4	47.557	63.882	97.76
**NLC Eveg 2**	8	86.464	45.476	38.07	49.558	110.798	156.4
**NLC Eveg 3**	8	93.028	35.904	61.21	71.688	104.24	153.1
**NLC Eveg 4**	8	103.18	52.479	41.65	61.888	139.515	184.54
**NLC Eveg 5**	8	90.629	58.283	45.95	57.025	92.538	227.86
**NLC Eveg 6**	8	102.884	27.784	61.75	87.447	118.285	148.69
**ANOVA P= 0.0521**							

Table no.12 Serum creatinine level in oophorectomized rat females in an experimental model of induced menopause

	N	Mean	Std. Dev.	Min	Item 25	Paragraph 75	Max
Normal control	8	0.356	0.044	0.295	0.343	0.361	0.446
Operated control	8	0.31	0.078	0.164	0.282	0.34	0.427
Control EG/PG	8	0.348	0.074	0.252	0.28	0.389	0.453
Control NN	8	0.406	0.154	0.2	0.357	0.442	0.695
NLC Eveg 1	8	0.355	0.062	0.248	0.344	0.387	0.425
NLC Eveg 2	8	0.329	0.105	0.1 79	0.233	0.414	0.454
NLC Eveg 3	8	0.397	0.074	0.282	0.367	0.409	0.545
NLC Eveg 4	8	0.39	0.069	0.317	0.347	0.431	0.517
NLC Eveg 5	8	0.416	0.042	0.351	0.388	0.44	0.479
NLC Eveg 6	8	0.341	0.054	0.268	0.291	0.377	0.417
ANOVA P= 0.165							

Table no.13 Serum urea level (mg/dL) in oophorectomised rat females in an experimental model of induced menopause

	N	Mean	Std. Dev.	Min	Item 25	Paragraph 75	Max
**Normal control**	8	40.201	10.457	25.43	31.208	46.028	56.53
**Operated control**	8	36.892	14.6	18.21	27.135	44.365	63.63
**Control EG/PG**	8	37.575	6.115	29.03	34.208	41.818	46.96
**Control NN**	8	39.842	11.996	22.5	29.96	47.87	53.38
**NLC Eveg 1**	8	39.165	6.562	28.52	36.635	41.445	48.7
**NLC Eveg 2**	8	38.054	5.342	29.6	35.847	39.158	48.07
**NLC Eveg 3**	8	40.664	10.943	23.03	34.845	47.41	57
**NLC Eveg 4**	8	40.325	10.584	18.99	38.017	48.04	51.11
**NLC Eveg 5**	8	31.119	8.017	16.7	27.798	35.048	41.35
**NLC Eveg 6**	8	41.77	6.434	34.54	37.818	42.91	54.91
**ANOVA P= 0.619**							

Table no. 14 The level of uricemia in females of the oophoroctomized rat in the experimental model of induced menopause

	N	Mean	Std. Dev.	Min	Item 25	Paragraph 75	Max
**Normal control**	8	1.321	0.436	0.589	0.99	1.58	1.912
**Operated control**	8	1.528	0.931	0.357	0.868	1.834	3.199
**Control EG/PG**	8	1.557	0.364	0.966	1.28	1.759	2.065
**Control NN**	8	1.544	0.774	0.816	1.114	1.669	3.149
**NLC Eveg 1**	8	0.78	0.153	0.595	0.621	0.885	0.984
**NLC Eveg 2**	8	1.526	0.539	0.766	1.233	1.797	2.327
**NLC Eveg 3**	8	1.464	0.56	0.839	0.969	1.749	2.308
**NLC Eveg 4**	8	1.45	0.511	0.721	1.105	1.804	2.213
**NLC Eveg 5**	8	1.247	0.964	0.407	0.713	1.246	3.429
**NLC Eveg 6**	8	1.247	0.513	0.417	0.87	1.621	1.879
**ANOVA P= 0.326**							

Table no.15 Level of serum acid phosphatase activity (U/L) in females of oophorectomized rats in an experimental model of induced menopause

	N	Mean	Std. Dev.	Min	Item 25	Paragraph 75	Max
**Normal control**	8	13.781	5.269	7.64	9.695	17.782	21.91
**Operated control**	8	15.39	5.71	7.77	12.703	17.683	27.07
**Control EG/PG**	8	12.601	4.079	4.82	10.838	15.147	17.31
**Control NN**	8	16.852	5.951	8.6	12.725	21.362	25.61
**NLC Eveg 1**	8	14.057	4.222	8.25	12.682	14.348	23.2
**NLC Eveg 2**	8	16.204	3.787	12.82	14.395	16.372	24.99
**NLC Eveg 3**	8	17.495	4.772	11.69	13.642	22.515	23.3
**NLC Eveg 4**	8	18.701	4.476	14.52	16.14	19.625	28.56
**NLC Eveg 5**	8	15.939	1.603	12.96	15.435	16.773	18.18
**NLC Eveg 6**	8	20.918	5.097	15.62	17.735	22.587	31.25
**ANOVA P = 0.0282**							

Table no.16 Total serum protein level (g/L) in oophorectomised rat females in an experimental model of induced menopause

	N	Mean	Std. Dev.	Min	Item 25	Paragraph 75	Max
**Normal control**	8	60.354	8.572	47.69	55.812	65.438	73.69
**Operated control**	8	48.015	3.51	43.74	46.11	49.472	54.57
**Control EG/PG**	8	60.136	12.931	43.64	49.603	69.878	79.07
**Control NN**	8	57.669	8.48	48.22	51.335	60.76	74.4
**NLC Eveg 1**	8	47.021	3.936	40.38	45.46	48.062	54.36
**NLC Eveg 2**	8	50.189	6.086	37.85	48.938	52.77	58.85
**NLC Eveg 3**	8	56.656	5.452	46.83	54.467	60.172	64.99

**NLC Eveg 4**	8	57.074	5.644	49.04	52.135	62.075	63.71
**NLC Eveg 5**	8	58.261	5.535	52.3	53.822	60.883	68.57
**NLC Eveg 6**	8	57.781	5.781	49.54	54.44	59.425	67.75
**ANOVA P= 0.000444 *****							

Table no. 17 Serum progesterone level in oophorectomised female rats with ovarian suppression experimental model of induced menopause

	N	Mean	Std. Dev.	Min	Item 25	Paragraph 75	Max
**Normal control**	8	44.474	27.771	21.81	34.855	38.885	106.05
**Operated control**	8	15.217	4.698	7.61	12.945	18.595	21.55
**Control EG/PG**	8	23.22	7.881	12.04	20.333	23.995	40.18
**Control NN**	8	22.39	11.827	10.95	13.232	28.575	44.72
**NLC Eveg 1**	8	18.585	9.353	6.6	12.503	24.678	31.75
**NLC Eveg 2**	8	14.213	3.385	10.73	11.528	16.008	19.53
**NLC Eveg 3**	8	18.3	5.517	11.51	12.608	23.108	24.85
**NLC Eveg 4**	8	20.011	9.789	10.66	14.178	20.452	42.5
**NLC Eveg 5**	8	13.025	3.521	8.29	9.968	15.235	17.84
**NLC Eveg 6**	8	27.959	37.371	10.42	12.642	17.55	120
**ANOVA P=0.0339 ***							

Table no. 18 Serum estradiol level in oophoroctomized rat females in experimental model of induced menopause

	N	Mean	Std. Dev.	Min	Item 25	Paragraph 75	Max
**Normal control**	8	31.95	19.822	13.84	24.115	29.99	74.96
**Operated control**	8	15.273	4.785	8.8	12.175	18.25	22.58
**Control EG/PG**	8	14.945	4.975	9.2	9.875	18.61	21.98
**Control NN**	8	16.375	10.589	9.1	10.465	15.445	41.65
**NLC Eveg 1**	8	16.033	4.324	9.6	13.6	19.127	21.23
**NLC Eveg 2**	8	15.107	3.989	9.7	12.825	16.78	21.16
**NLC Eveg 3**	8	13.066	3.551	9.1	9.75	14.895	18.37
**NLC Eveg 4**	8	11.918	4.157	9.1	9.6	12.807	21.37
**NLC Eveg 5**	8	10.82	1.315	9.5	9.825	12.14	12.72
**NLC Eveg 6**	8	15.499	5.077	9.8	9.875	20.112	21.31
				**ANOVA P=0.000527 *****			
